# Circulating nucleic acids: a new class of physiological mobile genetic elements

**DOI:** 10.12688/f1000research.7095.1

**Published:** 2015-09-30

**Authors:** Indraneel Mittra

**Affiliations:** 1 Translational Research Laboratory, Advanced Centre for Treatment, Research and Education in Cancer, Tata Memorial Centre, Navi-Mumbai, India

**Keywords:** Mobile genetic elements, horizontal gene transfer, circulating nucleic acids, circulating DNA, circulating chromatin, DNA damage, ageing

## Abstract

Mobile genetic elements play a major role in shaping biotic genomes and bringing about evolutionary transformations. Herein, a new class of mobile genetic elements is proposed in the form of circulating nucleic acids (CNAs) derived from the billions of cells that die in the body every day due to normal physiology and that act intra-corporeally. A recent study shows that CNAs can freely enter into healthy cells, integrate into their genomes by a unique mechanism and cause damage to their DNA. Being ubiquitous and continuously arising, CNA-induced DNA damage may be the underlying cause of ageing, ageing-related disabilities and the ultimate demise of the organism. Thus, DNA seems to act in the paradoxical roles of both preserver and destroyer of life. This new class of mobile genetic element may be relevant not only to multi-cellular organisms with established circulatory systems, but also to other multi-cellular organisms in which intra-corporeal mobility of nucleic acids may be mediated via the medium of extra-cellular fluid.

## Background

Barbara McClintock published her classic paper on mobile genetic elements (MGEs) in 1950 ^1^, but it took the scientific community several decades to appreciate the enormity of her discovery. Today, it is recognized that MGEs occur widely in nature in prokaryotes, archaea and eukaryotes and play a major role in shaping their genomes and bringing about evolutionary transformations and adaptation ^2– 5^. Their bizarre behavior of moving from one part of the genome to another distinguishes them from the functioning of conventional genetic elements.

MGEs belong to two classes *viz*, intra-genomic and inter-genomic. Intra-genomic MGEs are transposable elements (TEs) or transposons which constitute nearly 50% of the human genome but are variable among species and comprise 1%–5% of prokaryotic genomes ^6^. Inter-genomic transposable elements, on the other hand, underlie horizontal or lateral gene transfer (HGT) whereby segments of DNA are transferred from one organism to another ^7– 9^. Although HGT is known to occur extensively in bacteria and are responsible for development of antibiotic resistance ^10^, increasing evidence of HGT between other organisms is coming to light. For example, HGT between prokaryote and eukaryote, eukaryote and eukaryote, eukaryote and prokaryote has been reported ^7, 11^. Although the initial claims of presence of bacterial DNA in human genome were dismissed as erroneous ^12, 13^, recent evidence has confirmed the presence of bacteria DNA sequences in about one-third of healthy humans and in greater numbers in cancer cells ^14^. A recent analysis of public databases of transcriptome sequences of multiple organisms discovered that human beings have picked up at least 145 genes from other species during the course of evolution ^11^. Thus, HGT results in what is called a ‘web of life’ rather than a steadily bifurcating evolutionary tree ^8^.

## Circulating nucleic acids as a new class of mobile genetic elements

Based on a recent finding ^15^, a new class of mobile genetic elements is proposed *viz*, circulating nucleic acids, which are produced as a result of normal physiology and operate intra-corporeally or within the body of an organism. Circulating nucleic acids (CNAs) in the form of fragmented DNA and chromatin (DNAfs and Cfs) are known to circulate in blood and are derived from the hundreds of billions of cells that die through apoptosis in the adult human body on a daily basis ^16, 17^. These fragments have a size range of between 100bp–1000bp, have a half-life of 10–15 minutes and are ultimately removed by the liver ^18, 19^. The presence of Cfs (nucleosomes) in blood can be detected by a sandwich ELISA assay ^15^, but whether naked DNA circulates as such remains an open question since the possibility cannot be excluded that DNAfs isolated from plasma/serum are in fact products of the DNA purification process.

Results of a recent study summarized below have revealed that CNAs can act as mobile genetic elements ^15^. DNAfs and Cfs isolated from blood of healthy volunteers and cancer patients are actively taken up by cells in culture whereupon they rapidly accumulate in their nuclei and associate themselves with their chromosomes. The intracellular DNAfs and Cfs trigger a DNA-damage-repair-response (DDR) with up-regulation of multiple pathways of DNA damage and repair that facilitate their integration into host cell genome. Presence of human DNA in recipient mouse cell chromosomes could be detected by FISH while whole-genome sequencing uncovered tens of thousands of human reads in mouse cells. The integration of DNAfs and Cfs is stable and presence of extraneous DNA was demonstrable in single-cell clones developed from treated cells which had undergone numerous cell divisions. Genomic integration of DNAfs and Cfs results in phosphorylation of H2AX indicative of dsDNA breaks and up-regulation of apoptotic pathways in a proportion of cells. When injected intravenously into mice, DNAfs and Cfs integrate into cells of a variety of organs in the body, activate H2AX and the apoptotic marker active Caspase-3.

Whether genomic integration of CNAs occurs preferentially in a site-specific manner or is random is not known; but in either case, integration of CNAs would give rise to somatic mutations in the host genome. Since integration of CNAs occurred in all organs of the body examined ^15^, it may not be far-fetched to imagine that CNA-integration also occurs in germ cells. Genomic integration of CNAs would lead to DNA rearrangements, translocations and deletions ^20^ – changes that are hallmarks of ageing, and large DNA rearrangements and cell to cell variations in gene expression are typical of ageing cells ^21, 22^.

## CNAs integrate into host-cell genomes by a unique mechanism

According to the model depicted in the Figure, CNAs integrate into the genome by a unique mechanism in which activation of DDR plays a central role ^15^. When DNAfs and Cfs enter into a cell, the latter mistakenly perceives the intracellular DNAfs and Cfs with dsDNA breaks in their two ends as damaged “self” DNA and activates DDR even before DNA damage has actually occurred. The activated DDR joins up multiple disparate DNAfs and Cfs into long concatemers by non-homologous-end-joining as a part of the repair process. It is the integration into the host cell genomes of the concatemers by homologous or non-homologous recombination that brings about damage to DNA. Thus, paradoxically, the activation of DDR brings about damage to DNA rather than preserving DNA integrity. This model of DNA damage and repair in which DDR precedes DNA damage is the reverse of the classical model based on damage induced by ionizing and UV-radiations and chemicals wherein DDR is activated after DNA damage. It is possible that this model of DNA damage and repair that facilitates CNAs integration may apply to horizontally transferred DNA in other organisms in nature.

**Figure.**
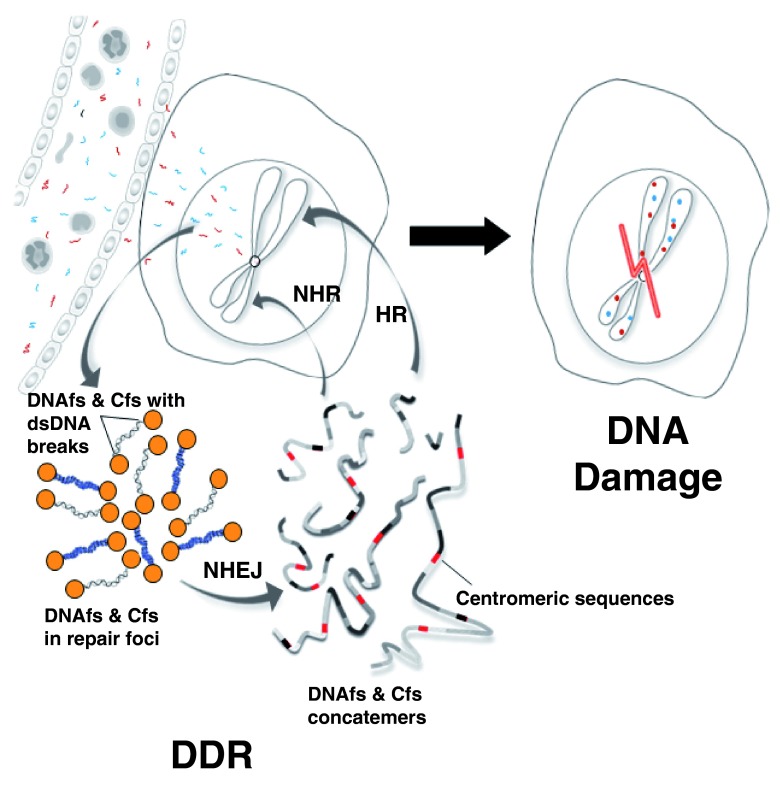
A new mechanistic model of genomic integration of CNAs in which DDR precedes DNA damage. NHEJ = non-homologous end-joining; HR = homologous recombination; NHR = non-homologous recombination. Reproduced with permission from Mittra *et al.*, J Biosci. 2015. **40**: 91–111.

## Implications

Although xenobiotics and DNA damaging agents constantly damage human DNA, these are usually transient and do not inflict permanent damage. CNAs, on the other hand, are ubiquitous, physiological and continuously arising, inflicting repeated damage and mutations to the somatic DNA. This naturally suggests that the somatic genome is not stable but remains in a state of turbulence characterized by DNA damage, mutations and rearrangements leading to DNA mosaicism and cell-to-cell variation in genomic structure and function. Indeed, cell-to-cell variations are being increasingly uncovered in the human body and are related to ageing ^21, 23– 25^. The above events and the accompanying genomic instability may give rise to cancerous transformations which is compatible with the steep rise in the incidence of cancer with increasing age ^26^. CNAs may also play an etiological role in several other disease conditions which are characterized by elevated levels of DNAfs and Cfs. These include auto-immune disorders ^27^, and a host of acute and chronic human pathologies, namely, sepsis ^28^, trauma ^29^, burns ^30^, organ transplantation ^31^, diabetes ^32^, myocardial infarction ^33^, stroke ^34^ and renal failure ^35^.

## Conclusions

CNAs are a new class of physiological, continuously arising intra-corporeal mutagenic agents that might be responsible for ageing, age-related disabilities and ultimately the demise of the organism. Thus, DNA seems to act in paradoxical roles of both preserver and destroyer of life. This new class of intra-corporeal mobile genetic elements may be relevant not only to multi-cellular organisms which have a developed circulatory system, but also to other multi-cellular organisms in which intra-corporeal mobility of CNAs may be mediated via the medium of extra-cellular fluid.

## References

[1] McClintock B: The origin and behavior of mutable loci in maize. *Proc Natl Acad Sci USA.* 1950;36(6): 344–55. 10.1073/pnas.36.6.344 15430309 PMC1063197

[2] Frost LS Leplae R Summers AO: Mobile genetic elements: the agents of open source evolution. *Nat Rev Microbiol.* 2005;3(9):722–732. 10.1038/nrmicro1235 16138100

[3] Deininger PL Moran JV Batzer MA: Mobile elements and mammalian genome evolution. *Curr Opin Genet Dev.* 2003;13(6):651–658. 10.1016/j.gde.2003.10.013 14638329

[4] Kazazian HH Jr: Mobile elements: drivers of genome evolution. *Science.* 2004;303(5664):1626–1632. 10.1126/science.1089670 15016989

[5] Brookfield JF: Evolutionary genetics: Mobile DNAs as sources of adaptive change? *Curr Biol.* 2004;14(9):R344–345. 10.1016/j.cub.2004.04.021 15120086

[6] Curcio MJ Derbyshire KM: The outs and ins of transposition: from mu to kangaroo. *Nat Rev Mol Cell Biol.* 2003;4(11):865–877. 10.1038/nrm1241 14682279

[7] Keeling PJ Palmer JD: Horizontal gene transfer in eukaryotic evolution. *Nat Rev Genet.* 2008;9(8):605–618. 10.1038/nrg2386 18591983

[8] Soucy SM Huang J Gogarten JP: Horizontal gene transfer: building the web of life. *Nat Rev Genet.* 2015;16(8):472–482. 10.1038/nrg3962 26184597

[9] Levin HL Moran JV: Dynamic interactions between transposable elements and their hosts. *Nat Rev Genet.* 2011;12(9):615–627. 10.1038/nrg3030 21850042 PMC3192332

[10] Polz MF Alm EJ Hanage W: Horizontal gene transfer and the evolution of bacterial and archaeal population structure. *Trends Genet.* 2013;29(3):170–175. 10.1016/j.tig.2012.12.006 23332119 PMC3760709

[11] Crisp A Boschetti C Perry M: Expression of multiple horizontally acquired genes is a hallmark of both vertebrate and invertebrate genomes. *Genome Biol.* 2015;16:50. 10.1186/s13059-015-0607-3 25785303 PMC4358723

[12] International Human Genome Sequencing Consortium, Adekoya E Ait-Zahra M: Initial sequencing and analysis of the human genome. *Nature.* 2001;409(6822):860–921. 10.1038/35057062 11237011

[13] Stanhope MJ Lupas A Italia MJ: Phylogenetic analyses do not support horizontal gene transfers from bacteria to vertebrates. *Nature.* 2001;411(6840):940–944. 10.1038/35082058 11418856

[14] Riley DR Sieber KB Robinson KM: Bacteria-human somatic cell lateral gene transfer is enriched in cancer samples. *PLoS Comput Biol.* 2013;9(6):e1003107. 10.1371/journal.pcbi.1003107 23840181 PMC3688693

[15] Mittra I Khare NK Raghuram GV: Circulating nucleic acids damage DNA of healthy cells by integrating into their genomes. *J Biosci.* 2015;40(1):91–111. 10.1007/s12038-015-9508-6 25740145 PMC5779614

[16] Fliedner TM Graessle D Paulsen C: Structure and function of bone marrow hemopoiesis: mechanisms of response to ionizing radiation exposure. *Cancer Biother Radiopharm.* 2002;17(4):405–426. 10.1089/108497802760363204 12396705

[17] van Nieuwenhuijze AE van Lopik T Smeenk RJ: Time between onset of apoptosis and release of nucleosomes from apoptotic cells: putative implications for systemic lupus erythematosus. *Ann Rheum Dis.* 2003;62(1):10–14. 10.1136/ard.62.1.10 12480662 PMC1754285

[18] Elshimali YI Khaddour H Sarkissyan M: The clinical utilization of circulating cell free DNA (CCFDNA) in blood of cancer patients. *Int J Mol Sci.* 2013;14(9):18925–18958. 10.3390/ijms140918925 24065096 PMC3794814

[19] Gauthier VJ Tyler LN Mannik M: Blood clearance kinetics and liver uptake of mononucleosomes in mice. *J Immunol.* 1996;156(3):1151–1156. 8557992

[20] Nevers P Saedler H: Transposable genetic elements as agents of gene instability and chromosomal rearrangements. *Nature.* 1977;268(5616):109–115. 10.1038/268109a0 339095

[21] Vijg J Dollé ME: Large genome rearrangements as a primary cause of aging. *Mech Ageing Dev.* 2002;123(8):907–915. 10.1016/S0047-6374(02)00028-3 12044939

[22] Bahar R Hartmann CH Rodriguez KA: Increased cell-to-cell variation in gene expression in ageing mouse heart. *Nature.* 2006;441(7096):1011–1014. 10.1038/nature04844 16791200

[23] Jacobs KB Yeager M Zhou W: Detectable clonal mosaicism and its relationship to aging and cancer. *Nat Genet.* 2012;44(6):651–658. 10.1038/ng.2270 22561519 PMC3372921

[24] Laurie CC Laurie CA Rice K: Detectable clonal mosaicism from birth to old age and its relationship to cancer. *Nat Genet.* 2012;44(6):642–650. 10.1038/ng.2271 22561516 PMC3366033

[25] McConnell MJ Lindberg MR Brennand KJ: Mosaic copy number variation in human neurons. *Science.* 2013;342(6158):632–637. 10.1126/science.1243472 24179226 PMC3975283

[26] Cancer Research UK. All Cancers (C00-C97 Excl. C44) Average Number of New Cases per Year and Age-Specific Incidence Rates, UK, 2009–2011. Reference Source

[27] Pisetsky DS Ullal AJ: The blood nucleome in the pathogenesis of SLE. *Autoimmun Rev.* 2010;10(1):35–37. 10.1016/j.autrev.2010.07.007 20659590 PMC3004144

[28] Rhodes A Wort SJ Thomas H: Plasma DNA concentration as a predictor of mortality and sepsis in critically ill patients. *Crit Care.* 2006;10(2):R60. 10.1186/cc4894 16613611 PMC1550922

[29] Lam NY Rainer TH Chan LY: Time course of early and late changes in plasma DNA in trauma patients. *Clin Chem.* 2003;49(8):1286–1291. 10.1373/49.8.1286 12881444

[30] Chiu TW Young R Chan LY: Plasma cell-free DNA as an indicator of severity of injury in burn patients. *Clin Chem Lab Med.* 2006;44(1):13–17. 10.1515/CCLM.2006.003 16375578

[31] Lui YY Woo KS Wang AY: Origin of plasma cell-free DNA after solid organ transplantation. *Clin Chem.* 2003;49(3):495–496. 10.1373/49.3.495 12600963

[32] Butt AN Shalchi Z Hamaoui K: Circulating nucleic acids and diabetic complications. *Ann NY Acad Sci.* 2006;1075:258–270. 10.1196/annals.1368.034 17108219

[33] Chang CP Chia RH Wu TL: Elevated cell-free serum DNA detected in patients with myocardial infarction. *Clin Chim Acta.* 2003;327(1–2):95–101. 10.1016/S0009-8981(02)00337-6 12482623

[34] Tsai NW Lin TK Chen SD: The value of serial plasma nuclear and mitochondrial DNA levels in patients with acute ischemic stroke. *Clin Chim Acta.* 2011;412(5–6):476–479. 10.1016/j.cca.2010.11.036 21130757

[35] Mittra I Nair NK Mishra PK: Nucleic acids in circulation: Are they harmful to the host? *J Biosci.* 2012;37(2):301–312. 10.1007/s12038-012-9192-8 22581336

