# Supplementary material for: Circulating nucleic acids: a new class of physiological mobile genetic elements
Source: F1000Res. 2015 Sep 30;4:924. [Version 1] doi: 10.12688/f1000research.7095.1 (PMC4654457; doi:10.12688/f1000research.7095.1)
Supplement: Peer review history

# Peer review: Referee response for version 1

**DOI:** 10.5256/f1000research.7639.r11047

**Published:** 2015-11-11

**Authors:** Manchanahalli Rangaswamy Satyanarayana Rao

**Affiliations:** 1 Molecular Biology and Genetics Unit, Jawaharlal Nehru Centre For Advanced Scientific Research, Bangalore, India

This article details a new paradigm in cancer genetics based on the recent original article published by the same group. The idea of the origin of the cancer phenotype based on the circulating nucleic acid/ chromatin fragments is really thought provoking and will generate a lot of interest in the cancer researchers. The manuscript is well written summarizing the salient features of their recently published article.

I have read this submission. I believe that I have an appropriate level of expertise to confirm that it is of an acceptable scientific standard.

# Peer review: Referee response for version 1

**DOI:** 10.5256/f1000research.7639.r10595

**Published:** 2015-10-13

**Authors:** Silvia Gravina

**Affiliations:** 1 Department of Genetics, Albert Einstein College of Medicine, Bronx, NY, USA

In this Opinion Article entitled “ Circulating nucleic acids: a new class of physiological mobile genetic elements”, Indraneel Mittra investigated the topic of circulating nucleic acids as a new class of continuously arising DNA damaging agents.

The author discusses a recent work (Mittra *et al*., 2015) which describes how free nucleic acids not only can enter cell nuclei freely, but they integrate in the host genome and activate proteins of DDR and apoptotic pathways. In particular, in the Opinion Article, the author hypothesizes that cell free DNA continuously arising induced damage can be the cause of aging and age related diseases.

The manuscript is certainly well written and attractive, and discusses a very novel yet important topic in the aging field. My only concern is that in two parts of the manuscript the author jumps to conclusions too quickly, and I would suggest rephrasing certain statements.

More specifically:

 

- pg2, mid right; “Whether genomic integration of CNAs occurs preferentially in a site-specific manner or is random is not known; but in either case, integration of CNAs would give rise to somatic mutations in the host genome.”
  This has to be demonstrated yet. We don’t really know if CNA integration is a direct cause of somatic mutations in the host genome. I would suggest making this statement milder (i.e.” Whether genomic integration of CNAs occurs preferentially in a site-specific manner or is random is not known. It is reasonable to speculate that integration of CNAs may give rise to somatic mutations in the host genome.” or “a fascinating hypothesis yet to be tested is that that integration of CNAs may give rise to somatic mutations in the host genome”.)
- Same for pg2 bottom part; “CNAs, on the other hand, are ubiquitous, physiological and continuously arising, inflicting repeated damage and mutations to the somatic DNA”. I would suggest here-as well- a milder statement (i.e., CNAs, on the other hand, are ubiquitous, physiological and continuously arising, inflicting repeated damage and potentially causing mutations to the somatic DNA”).

I have read this submission. I believe that I have an appropriate level of expertise to confirm that it is of an acceptable scientific standard.
